# Dietary complexity and hidden costs of prey switching in a generalist top predator

**DOI:** 10.1002/ece3.6375

**Published:** 2020-05-27

**Authors:** Rosemary J. Moorhouse‐Gann, Eleanor F. Kean, Gareth Parry, Sonia Valladares, Elizabeth A. Chadwick

**Affiliations:** ^1^ School of Biosciences Cardiff University Cardiff UK; ^2^ Durrell Wildlife Conservation Trust, Trinity Jersey UK; ^3^ Gloucestershire Wildlife Trust The Conservation Centre Gloucester UK

**Keywords:** body condition score, fish communities, foraging strategies, freshwater food web, multi‐model inference, mustelid, predator–prey interactions, wildlife diet analysis

## Abstract

1. Variation in predator diet is a critical aspect of food web stability, health, and population dynamics of predator/ prey communities. Quantifying diet, particularly among cryptic species, is extremely challenging, however, and differentiation between demographic subsets of populations is often overlooked.
2. We used prey remains and data taken *postmortem* from otter *Lutra lutra* to determine the extent to which dietary variation in a top predator was associated with biotic, spatial, and temporal factors.
3. Biotic data (e.g., sex, weight, and length) and stomach contents were taken from 610 otters found dead across England and Wales between 1994 and 2010. Prey remains were identified to species where possible, using published keys and reference materials. Multi‐model inference followed by model prediction was applied to test for and visualize the nature of associations.
4. Evidence for widespread decline in the consumption of eels (*Anguilla anguilla*) reflected known eel population declines. An association between eel consumption and otter body condition suggested negative consequences for otter nutrition. Consumption of *Cottus gobio* and stickleback spp. increased, but was unlikely to compensate (there was no association with body condition). More otters with empty stomachs were found over time. Otter sex, body length, and age‐class were important biotic predictors of the prey species found, and season, region, and distance from the coast were important abiotic predictors.
5. Our study is unique in its multivariate nature, broad spatial scale, and long‐term dataset. Inclusion of biotic data allowed us to reveal important differences in costs and benefits of different prey types, and differences between demographic subsets of the population, overlaid on spatial and temporal variation. Such complexities in otter diet are likely to be paralleled in other predators, and detailed characterization of diet should not be overlooked in efforts to conserve wild populations.

## Graphical abstract

Prey remains collected postmortem from 610 otters found dead in the UK reveal a diverse diet that has changed over time between 1994 and 2010. Opportunistic prey switching (e.g., from eel to bullhead) may support population resilience, but associations with body condition suggest negative consequences. Detailed characterization of diet is an important, but sometimes overlooked, aspect of conservation management.

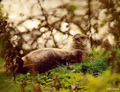

## 1 INTRODUCTION

Dietary strategies are highly variable, on a broad spectrum between specialism and generalism (Bolnick et al., 2003; Futuyma & Moreno, 1988). Generalist consumers often have the capacity to switch to alternative food sources when their preferred dietary items are unavailable (Almeida et al., 2012; Fox, 2005; Kjellander & Nordstrom, 2003). Such dietary plasticity decreases a species’ sensitivity to change and stabilize food webs (van Baalen, Krivan, van Rijn, & Sabelis, 2001; Clare, 2014). Despite generalists being more robust to change, there may still be costs associated with dietary switching. For example, switching to suboptimal prey with a lower nutritional value or a higher contaminant load could reduce the fitness of a species in the short term (Ruiz‐Olmo & Jimenez, 2009). In the longer term, consequences for population dynamics might threaten persistence of the species (Korpimaki, 1992; Schweiger, Funfstuck, & Beierkuhnlein, 2015), with implications for food web stability and ecosystem functioning (Spitz & Jouma'a, 2013).

Carnivore diet reflects both temporal and spatial variation in prey availability (Elmhagen, Tannerfeldt, Verucci, & Angerbjorn, 2000; Prugh, Arthur, & Ritland, 2008; Virgos, Llorente, & Cortes, 1999). Availability of prey relates not only to abundance in the environment, but also to ease of capture, which varies with species (Sih & Christensen, 2001) and time of year (Krawczyk, Bogdziewicz, Majkowska, & Glazaczow, 2016). Variation in feeding behavior may result from individual variation in morphology, health, and competition, among other variables (e.g., Araujo, Bolnick, Martinelli, Giaretta, & dos Reis, 2009; Beck, Iverson, Bowen, & Blanchard, 2007; Bolnick, 2004; Broekhuis, Thuo, & Hayward, 2018; Kitchener, 1999; McDonald, 2002; Murray, Edwards, Abercrombie, & St. Clair, 2015; Svanback & Bolnick, 2007; Woo, Elliott, Davidson, Gaston, & Davoren, 2008). In addition, dietary requirements may vary between demographic subsets of a population including between age‐classes and sexes (Houston et al., 2007; Magnusdottir, Stefansson, von Schmalensee, Macdonald, & Hersteinsson, 2012; Navarro et al., 2009; Riccialdelli et al., 2013), which can lead to niche partitioning. The consequences of dietary switching may therefore differ significantly between individuals and groups, which may result in exposure to differing selection pressures including contaminant and parasite loads.

A variety of methods are available for studying the diet of animals, each with associated strengths and weaknesses. Noninvasive analysis of fecal samples using morphological or molecular methods predominates (Pompanon et al., 2012; Ripple, Beschta, Fortin, & Robbins, 2014; Valenzuela, Rey, Fasola, & Schiavini, 2013). Using this strategy, it is difficult to collect information about the individual, unless the animal is captured or DNA analyses are employed (Dunn et al., 2018; Prugh et al., 2008), which might provide valuable insight into dietary variation. Consequently, it is difficult to detect associations between diet and biotic variation. To achieve this, samples and data collected postmortem offer a unique opportunity to link the diet of individuals to a range of biotic variables (Lanszki, Bauer‐Haaz, Szeles, & Heltai, 2015).

The Eurasian otter (*Lutra lutra,* hereafter referred to as “otter”) is a semi‐aquatic carnivore specialized to feed in aquatic ecosystems, mainly on fish (Almeida et al., 2012; Britton, Pegg, Shepherd, & Toms, 2006; Krawczyk et al., 2016; Kruuk, 1995). Otter numbers declined dramatically across much of their European range during the last century. This decline was thought to be due to the bioaccumulation of pesticides and polychlorinated biphenyls (PCBs) and habitat modification (reviewed in Chanin, 2003). Population distributions have since generally increased significantly across western Europe (Mason & Macdonald, 2004; Roos, Loy, de Silva, Hajkova, & Zemanová, 2015) but those recoveries are occurring in an environment that has changed considerably in the intervening decades, with respect to habitat, prey availability (Hayhow et al., 2016), and freshwater pollution (Murray, Thomas, & Bodour, 2010). These factors are likely to play significant roles in determining the rate of recovery and changing distribution of recovering species (Hayes & Harestad, 2000).

In the current study, we utilized a biobank of samples and data collected from otters found dead in England and Wales over 16 years, to test how diet varied between individuals with differing biotic characteristics (such as sex, age, and condition), while controlling for temporal and spatial variation.

## 2 METHODS

### 2.1 Sample collection

Otters found dead (mostly as a result of road traffic accident, RTA [92%]) were collected across England and Wales (see Figure 5, Section 3) between 1994 and 2010, as part of a national scheme coordinated by Cardiff University. The entire stomach and intestine were taken during postmortem examination of 610 otters and frozen at −20°C.

### 2.2 Abiotic variables

The year, month, and location where each otter was found were recorded. Location data were provided as national grid references accurate to the nearest 100 m and were used to define region and distance to the coast. Distance to the coast via the nearest river (river distance) was measured using ArcMap GIS (V.9.2, ESRI, 2006). Where otters were found more than 1,000 m from a river, river distance was omitted. Each otter was assigned to one of eight regions, based on aggregations of adjacent river catchments (see Figure 5, Section 3).

### 2.3 Biotic variables

Each otter was examined in detail at postmortem; sex, age‐class (adult, subadult, and juvenile), reproductive status (females only), weight (kg, to the nearest 10 grams), length (mm, to the nearest 5 mm, measured nose to anus and anus to tail tip), and cause of death were determined. Morphometric and reproductive data were used to assign age‐class, such that males below 3 kg and females below 2.1 kg are recorded as juveniles. Above these weights, otters are considered adult if reproductively mature (males with baculum length ≥ 60 mm (van Bree, Jensen, & Kleijn, 1966), females with signs of reproductive activity such as placental scarring and prominent teats) and subadult if reproductive maturity is not apparent. Based on examination of the uterus and teats, female reproductive status was categorized as never reproduced, quiescent, pregnant, or lactating. Length and weight were used to calculate a condition index (*K* = *W*/*aL^n^*, where *W* = total body weight in kg, *L* = total length in m; for males *a* = 5.87 and *n* = 2.39; for females *a* = 5.02 and *n* = 2.33, Kruuk, Conroy, & Moorhouse, 1987).

### 2.4 Dietary analysis

Stomach and intestine samples were defrosted and rinsed through a fine sieve. Recognizable dietary items (e.g., feathers and fur) were recorded. Remaining solid material was transferred to a liquid detergent solution (water:detergent, 10:1) to aid removal of soft tissues. Samples were filtered repeatedly, and detergent solution replaced until only hard prey items remained. After air‐drying, prey items were examined using a binocular microscope (Leica 2000, x7‐x30) and identified with the aid of published keys (Cham, 2007; Conroy, Watt, Webb, & Jones, 2005; Day, 1966; Miranda & Escala, 2002; Teerink, 1991) and by comparison with reference material. Prey items were identified to species level whenever possible but to reliably differentiate salmon and trout, recovery of the atlas bone or jaw parts was required and these suffered poor recovery rates. Similarly, cyprinids were not distinguishable to species from their vertebrae but where pharyngeal bones were found species identification was made. These data were reported as frequency of occurrence (FO = *t*/*n*, where *t* = total number of occurrences of a particular prey type and *n* = total number of samples examined) and as relative frequency of occurrence (RFO = *t*/*p*, where t = total number of occurrences of a particular prey type and *p* = total number of occurrences of all prey types) to enable comparison with earlier studies (Clavero, Prenda, & Delibes, 2004). RFO was calculated both relative to all prey types across taxa and (for fish) relative to total occurrences of all fish prey types. For statistical analyses, the response variable was the presence/absence of either a specified prey type or an empty stomach.

### 2.5 Statistical analyses

To identify the importance of biotic and abiotic associations with otter diet, we used a series of generalized additive models (GAMs) in the R software environment (version 3.3.1, R Core Team, 2016) utilizing the mgcv (Wood, 2011) package, with binomial error distributions (see Table 1 for global model specifications, including all independent variables).

**TABLE 1:** Independent variables included in global Model Groups 1–4

Independent variable	Categories/data range	Included in Model Group			
		1	2	3	4
Biotic variables					
Sex (f)	Female, Male.	y	—	y	—
Age‐class (f)	Adult, subadult, juvenile. NB. Juveniles excluded from Model Groups 3 and 4	y	—	y	y
Body length (c)	490–1,307 mm	y	y	y	y
Body condition (c)	0.5–2	y	y	y	y
Reproductive status (f)	Never reproduced, quiescent, pregnant or lactating. Term applicable to females only	—	y	—	y
Cause of death (f)	Sudden (*n* = 560), ill (*n* = 40), unknown (*n* = 10)	y	y	—*	—*
Biotic interactions					
Sex: Age, Sex: Body length, Sex: Body condition, Age: Body length, Age: Body condition, Body length: Body condition		y	—	y	—
Reproductive status: Body condition, Reproductive status: Body length		—	—	—	y
Abiotic variables					
Year (c) Month (c)	Month (Jan–Dec), fitted with circular spline, nested in Year	y	y	y	y
Year (c)	1994–2010	y	y	y	y
Region (f)	8 regions (see Figure 5)	y	y	y	y
Distance from the coast (c)	Distance to coast, following river channel, 0–235 km).	y	y	y	y

Note

Factors (categories) and continuous variables are denoted (f) and (c), respectively. Model Groups 1 and 2: Dependent variable is presence/absence of an empty stomach. Model Groups 3 and 4: Dependent variable is the presence of each of 11 different prey types. Prey types were as follows: Eel, bullhead, cyprinid, salmonid, stickleback, crustacean, mammal, bird, insect, amphibian, and marine fish. Cause of death was not included in Model Groups 3 and 4 due to the vast majority of deaths being “sudden” in these reduced datasets (93.8% for Model Group 3, and 92.0% for Model Group 4). The link functions selected were as follows: probit for eel Model Group 3 and bullhead Model Group 4; cloglog for bullhead Model Group 3 and crustacean Model Group 4; logit for cyprinid Model Group 4; cauchit for all remaining models.

First, we examined whether or not otters had recently fed on vertebrate prey, using presence/absence of prey remains (of any type) in the gut as the binomial dependent variable, and tested for associations with a range of biotic and abiotic independent variables (as specified in Table 1, Model Groups 1 and 2). Model Group 1 used all individuals (*n* = 610) and included sex as an independent variable so that we could test for sexual differentiation, while Model Group 2 (*n* = 268) excluded males and additionally included female reproductive status as an independent variable. Age‐class and the majority of biotic interactions were excluded from Model Group 2 due to the reduced sample size (see Table 1).

Secondly, for those otters where identifiable prey remains were found, we used presence/absence of each of 11 individual prey types as the binomial dependent variable, and tested for associations with biotic and abiotic variables (as specified in Table 1, Model Groups 3 and 4). “Prey type” was either species (European eel *Anguilla anguilla*, European bullhead *Cottus gobio*), or broader taxonomic/functional groupings where species identification was unreliable or where presence data for individual species was too few to permit robust modelling (cyprinid, salmonid, stickleback (family Gasterosteidae), crustacean, mammal, bird, insect, amphibian, and marine fish). For these Model Groups, we excluded juveniles due to small sample size (only 4 individuals with prey remains). Model Group 3 included all remaining individuals (*n* = 501) and included sex as an independent variable, while Model Group 4 (*n* = 226) further excluded males and included female reproductive status (as for Model Group 2).

In all Model Groups (1–4), interaction terms were included for biotic terms if there was an appropriate biological hypothesis and sufficient data to allow robust model predictions (as specified in Table 1). The most appropriate link function for each model was selected based on the lowest AIC (Akaike information criterion) values.

Interpretation of the most important variables was achieved using a multi‐model inference approach, using the dredge function in MuMIn (Bartoń, 2018) and applying model averaging where delta AICc (AIC corrected for small sample size) <2 (Symonds & Moussalli, 2011). If the AICc of the second best model was >2, a model‐averaging approach was not applied and the results from the single best model were reported. Rather than reliance on *p*‐values alone, the most important relationships were described: those which either appeared in all top models (relative importance [RI] = 1) regardless of probability, or where RI was >0.5 *and* the relationship was statistically significant (*p* < .05). Note that in some cases, variables were deemed important by inclusion in multiple (or all) top models, even where model averaging suggested nonsignificance (*p* < .05; Burnham & Anderson, 2002). All global model residuals were visually assessed for temporal autocorrelation (using the autocorrelation function “acf” in R, with a maximum lag of 12 months). There was no evidence for significant autocorrelation in the residuals from any global models, so no further adjustments were made to model structures.

For model predictions (using the “predict” function), we modelled the effect of each selected variable, while controlling all other important variables to either the most frequently observed level (Age = adult, Sex = male, cause of death = sudden, Year = 2008, Month = January and Region = Wales) or to their median Condition = 1, Length = 1,050 mm, river distance = 30 km. Where predictions were calculated for different age‐classes, body length was adjusted to the median specific to that group (juvenile: 663 mm, subadult: 1,005 mm, adult: 1,105 mm).

## 3 RESULTS

Of the 610 otters examined, 268 were female (129 = never reproduced, 44 = pregnant or lactating, 90 = quiescent, 5 = not possible to assess) and 342 were male. Most were adult (343) or subadult (251) with very few juvenile (15), and it was not possible to determine the age‐class of one individual. Most died suddenly, for example, by road traffic accident (*n* = 560), and some died of ill health (*n* = 40) while in other cases cause of death was unknown (*n* = 10).

Of all 610 otter gut samples examined, 505 (82.79%) contained identifiable vertebrate prey remains. Fish were the main component of otter diet (71.18% RFO). Amphibians also made a substantial contribution (14.86% RFO), while insect, mammal, bird, and crustacean remains were relatively rare (≤5% RFO; Table 2). Of the fish, the most common identified species were bullhead (22.59% RFO) and eel (17.61% RFO). Cyprinids were commonly found (22.73% RFO) but note that this total potentially encompasses 16 species found in the UK, of which 9 were definitively identified (Table 2). Salmonids were commonly found (21.88% RFO), and where identification was considered reliable most remains were of trout (Table 2). Sticklebacks were also frequently found (11.36%), but in most cases it was not possible to discriminate between three‐spined (*Gasterosteus aculeatus*) and nine‐spined stickleback (*Pungitius pungitius*). Most species found were freshwater, with only 3.83% of samples including remains of marine fish. Fish remains identified as family Percidae, stone loach (*Barbatula barbatula*), and pike (*Esox lucius*) were also found occasionally but were not included in analyses due to low sample size (*n* = 18, 14, 3).

**TABLE 2:** Prey remains identified from otter stomachs

*z*	RFO/fish	RFO/all	FO (*n*)	Species names/ other taxonomic subgroupings identified
Anguillidae	18	13	20 (124)	Eel *Anguilla anguilla*
Cottidae	23	16	26 (159)	Bullhead *Cottus gobio*
Cyprinid	23	16	26 (160)	Minnow *Phoxinus phoxinus* (54), chub *Leuciscus cephalus* (12), roach *Rutililus rutilus* (11), dace *Leuciscus leuciscus* (4), carp *Carassius* spp.(3), tench *Tinca tinca* (2), barbel *Barbus barbus* (2), common bream *Abramis brama* (2), rudd *Scardinius erythrophthalamus* (1)
Salmonid	22	16	25 (154)	Salmon *Salmo salar* (3), trout *Salmo trutta* (38)
Stickleback	11	8	13 (80)	3‐spined stickleback *Gasterosteus aculeatus* (2)
Marine fish	4	3	4 (27)	Goby (15) (family Gobiidae), flatfish (17: including dab *Limanda limanda* [1], Flounder *Platichthys flesus* [2], plaice *Pleuronectes platessa* [5], brill *Scophthalmus rhombus* [1]), four‐bearded rockling *Enchelyopus cimbrius* (5), mackerel (1), Family blennidae (4), wrasse (family Labridae, seven species found in UK waters) (3), sand eel (1)
Amphibian	NA	15	24 (147)	Frog *Rana temporaria* (60), Toad *Bufo spp* (1), Newt *Lissotriton spp* (11) (in UK only *L. vulgaris* or *L. helveticus*)
Crustacean	NA	3	5 (28)	Crayfish (16), crab (4), mollusk (9) (not identified to species)
Mammal	NA	3	5 (32)	Common shrew *Sorex araneus* (2), water shrew *Neomys fodiens* (1), *Sorex* spp (4), rabbit *Oryctolagus cuniculus* (3), wood mouse *Apodemus sylvaticus* (2)
Bird	NA	3	5 (31)	Rallidae (includes water rail *Rallus aquaticus*, moorhen *Gallinula chloropus*) and coot *Fulica atra* (4), also Anseriforme spp (diverse family of waterfowl, including ducks, geese, swans) (3)
Insect	NA	5	8 (47)	Diptera (true flies) (1), Dytiscus (diving beetle) (5), Odonata (includes dragonflies and damselflies) (3).
Other fish				Percidae (18), stone loach (*Barbatula barbatula*) (14) and pike (*Esox lucius*) (3)

Note

The 11 prey types represent species, or higher taxonomic ranks, as used in statistical analyses. RFO = relative frequency of occurrence (total occurrences of a particular prey type/total number of occurrences for all prey types, calculated both relative to the total occurrence of fish prey (*n* = 704, “/fish”), and to all prey (*n* = 989, “/all”). FO = frequency of occurrence (total occurrences of a particular prey type/total number of samples (*n* = 610)), number of individuals in which that prey type occurred is indicated in brackets (*n*).

### 3.1 Generalized additive models: presence/ absence of prey remains

The absence of vertebrate prey remains (i.e., the likelihood of having an empty stomach at death) was significantly associated with both biotic and abiotic variables. For Model Group 1 (analysis including both sexes), after applying the dredge function to our global model, there was only one top model (deviance explained = 22.7%, Appendix 1: Table A1). Juveniles were approximately seven times more likely to have empty stomachs than adults (*z* = 3.01, *p* = .003) or subadults (*z* = 3.34, *p* = <.001; model predictions: juvenile = 0.85 ± 0.08; subadult = 0.125 ± 0.026; adult = 0.118 ± 0.024). Males were significantly more likely to have empty stomachs than females (*z* = 2.48, *p* = .013, model predictions: females = 0.068 ± 0.014; males = 0.113 ± 0.025). Absence of prey was associated with body condition, but the nature of the association differed with body size (significant length: condition interaction, *z* = 2.49, *p* = .013). For larger otters, those with empty stomachs tended to be in poorer condition, but this association was not apparent for smaller otters. Feeding was not significantly associated with reproductive status.

**FIGURE 1:**
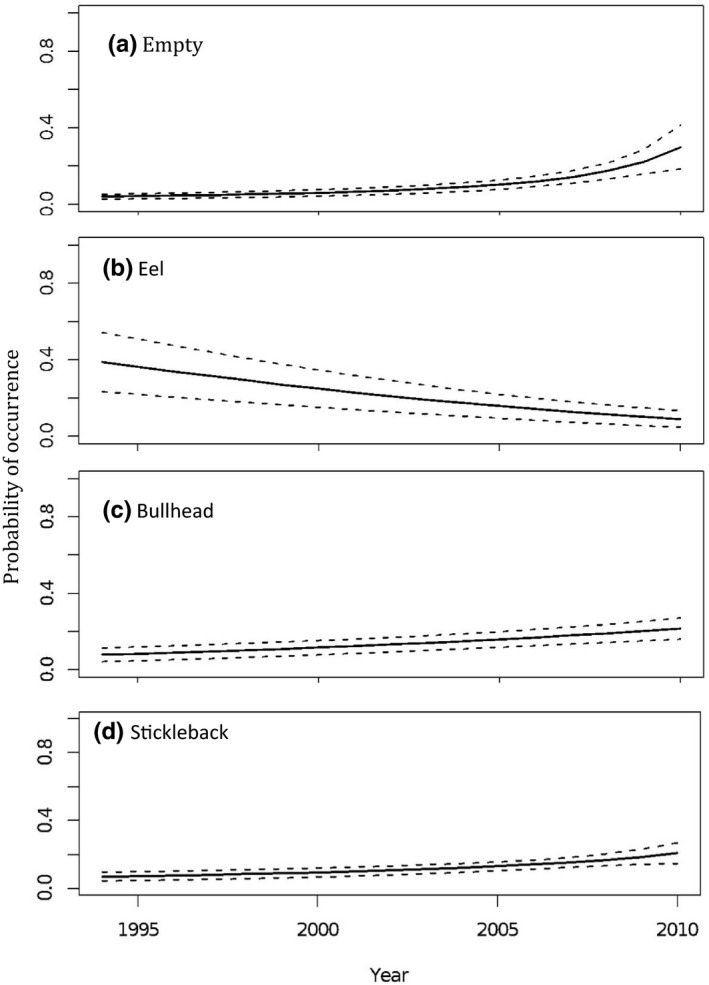
Temporal trends in otter diet between 1994 and 2010. Plot (a) indicates the change in the probability of otters having empty stomachs with time. Plots (b), (c), and (d) indicate changes in the probability of finding eel, bullhead, and stickleback remains, respectively, in otter stomachs over time. Model predictions are plotted alongside the standard error surrounding those predictions. For the associations between year and other prey types, refer to Appendix 1: Table A2

Between 1994 and 2010, the likelihood of having an empty stomach increased significantly (*z* = 2.59, *p* = .01), with a probability of an empty stomach at 0.037 ± 0.01 in 1994, increasing to 0.215 ± 0.075 in 2010 (Figure 1a). Otters that died following illness were almost four times more likely to have empty stomachs than those that died suddenly (*z* = −2.99, *p* = .003, model predictions: sudden = 0.113 ± 0.0248; ill = 0.445 ± 0.248). There was a small but significant effect of distance from the coast, with otters found in coastal areas more likely to have empty stomachs (*z* = −2.43, *p* = .015), varying between a probability of 0.145 ± 0.038 at the coast to 0.118 ± 0.026 30 km inland, and to 0.071 ± 0.017 135 km inland (note that 89% of otters for which it was possible to measure distance from the coast [*n* = 506] were found within 135 km of the coast, with 50% <30 km from the coast).

### 3.2 Generalized additive models: stomachs containing prey remains

All 11 averaged models retained both abiotic and biotic variables, indicating that both are important for understanding otter diet. Below we describe the most important relationships (defined using RI (relative importance) and p (probability) as specified in Methods).

#### 3.2.1 Biotic variables

**FIGURE 2:**
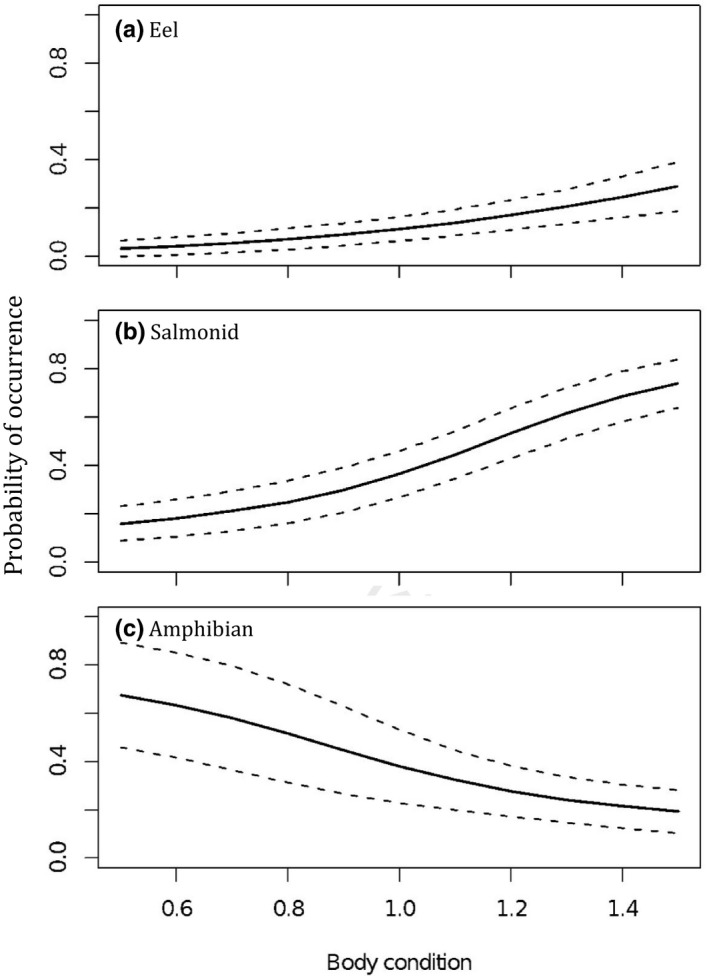
Variation in otter diet with body condition. Associations between otter body condition index and different prey remains: (a) eel, (b) salmonid, and (c) amphibian. Model predictions are plotted alongside the standard error surrounding those predictions. For the associations between body condition and other prey types, refer to Appendix 1: Table A2

Otter body condition, body length, age‐class, and sex were all important predictors of otter diet (Appendix 1: Table A2). Reproductive status was not an important predictor of diet, so Model Group 4 is not discussed further and all results described below are taken from Model Group 3 (including both sexes). The likelihood of consuming amphibians and mammals was negatively associated with body condition (amphibian: *z* = 1.96, RI = 0.85, *p* = .05; mammal: *z* = 2.05, RI = 1, *p* = .041). For eels and salmonids, the reverse was true (eel: *z* = 2.23, RI = 1, *p* = .026; salmonid: *z* = 2.12, RI = 1, *p* = .034; Figure 2).

**FIGURE 3:**
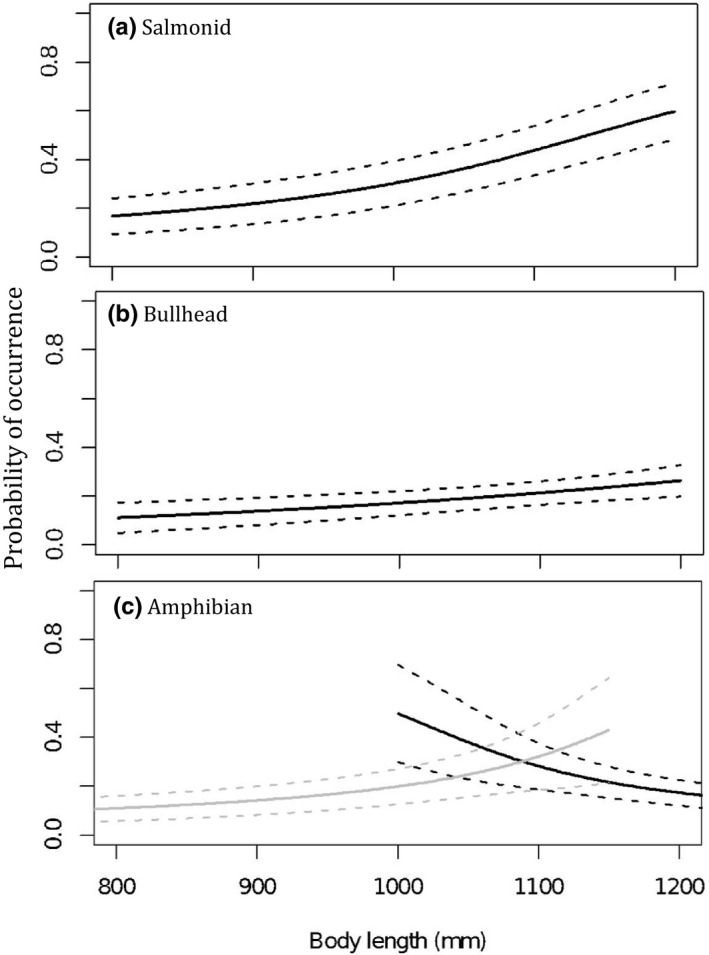
Dietary associations with otter body length. Associations between otter body length and different prey remains: (a) salmonid, (b) bullhead, and (c) amphibian (plotted separately for adults (black) and subadults (gray)). Model predictions are plotted alongside the standard error surrounding those predictions. For the associations between body length and other prey types, refer to Appendix 1: Table A2

Larger otters (those with greater body length) were more likely to have consumed salmonids (*z* = 2.07, RI = 1, *p* = .039) and bullhead (*z* = 2.043, RI = 1, *p* = .041). For predation on amphibians, the association with body length was dependent on age: Adult otters showed a negative association between length and consumption (i.e., large adults were less likely to eat amphibians), whereas subadult otters showed the reverse (large subadults were more likely to eat amphibians (RI = 1, *p* = .006; Figure 3)). The likelihood of eating crustaceans decreased with length but the effect size was very small and on average this term was not statistically significant (*z* = 1.34, RI = 1, *p* = .18).

Sex proved to be an important (although statistically insignificant) variable in explaining the prevalence of some prey types. Female otters were 10% more likely than males to prey on bullhead (*z* = 0.44, RI = 1, *p* = .66, model predictions: females = 0.299 ± 0.063; males = 0.191 ± 0.047). The opposite trend was observed for cyprinid consumption, with males being >20% more likely to have consumed this group than females (*z* = 0.97, RI = 1, *p* = .33, model predictions: females = 0.304 ± 0.081; males = 0.513 ± 0.117). Predation on mammals was rare, and sex differences varied with age (the sex: age‐class interaction was important, although not significant, RI = 1, *p* = .22). Adult males were more likely to prey on mammals than subadult males (predictions: adult males = 0.084 ± 0.093; subadult males: = 0.027 ± 0.010), whereas for females likelihood was low in both age groups (predictions: adult females = 0.017 ± 0.012; subadult females = 0.038 ± 0.015).

#### 3.2.2 Abiotic variables

All abiotic variables tested (month, year, region, and distance from the coast) were important predictors of otter diet (Appendix 1: Table A2).

**FIGURE 4:**
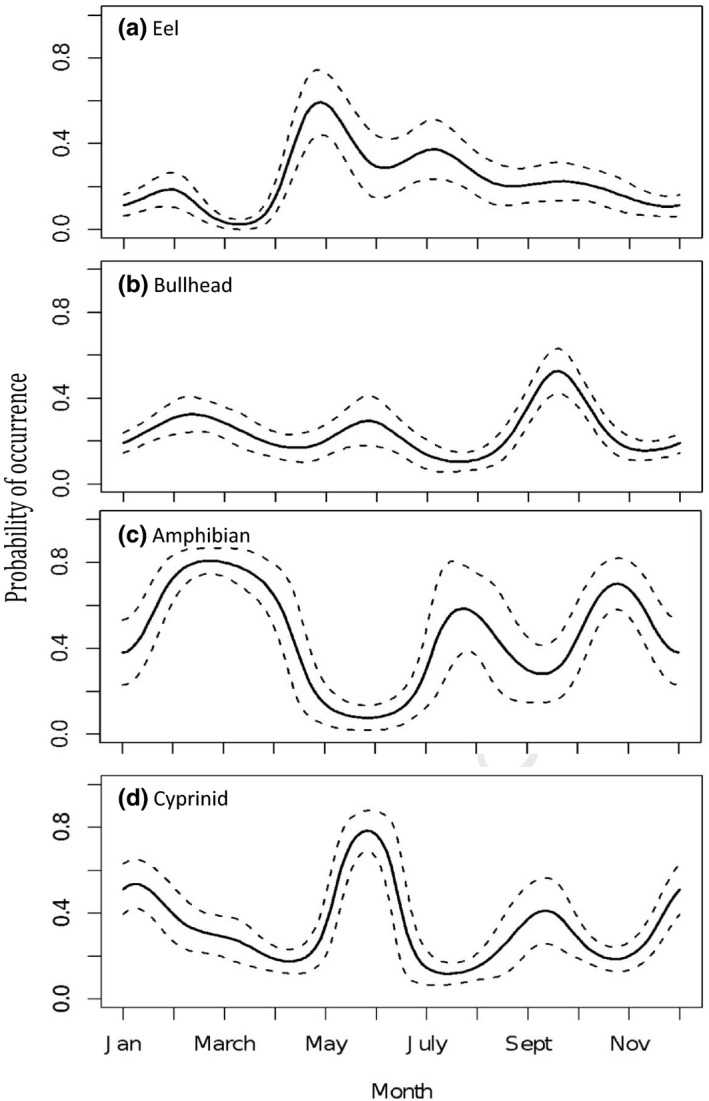
Seasonality in otter diet. Seasonality of the prevalence of different prey remains: (a) eel, (b) bullhead, (c) amphibian, and (d) cyprinid. Model predictions are plotted alongside the standard error surrounding those predictions. Details for crustacean predation not shown due to a low probability of occurrence and large standard errors. For the associations between month and other prey types, refer to Appendix 1: Table A2

Seasonality (month nested within year) appeared in all top models for eel, bullhead, amphibian, cyprinid, and crustaceans (RI = 1). Eel consumption rose suddenly from near zero in March to a major peak in May followed by a steady fall through summer and autumn (Figure 4a). Bullhead were predated throughout the year, but with a clear peak in autumn (Figure 4b). Consumption of amphibians showed a clear trough in summer; the highest peak was in spring, but autumn and winter consumption was also high (Figure 4c). Cyprinid predation was highly seasonally variable, with the largest peak in early summer and another high peak in December–January and smaller peak in autumn (although note larger error around autumn peak; Figure 4d).

**FIGURE 5:**
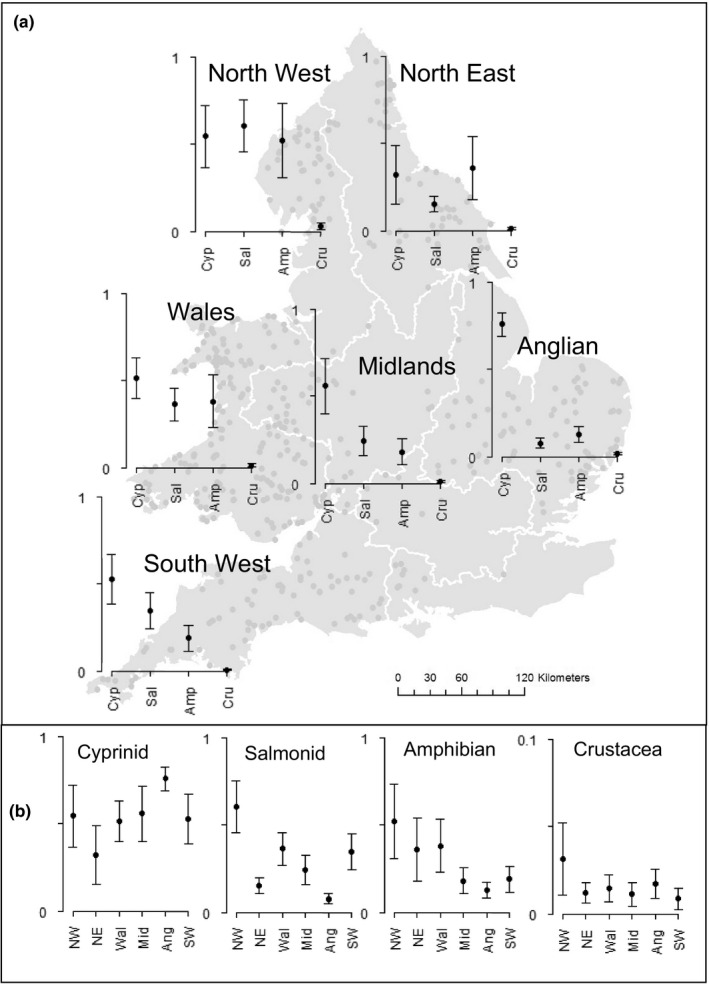
Spatial variation in otter diet. Black circles indicate average model‐predicted probability of prey taxon occurring in otter diet (± standard error), for all models where significant associations with region were indicated. Note that other prey types, for example, eel and bullhead, were equally important across all regions (see frequency of occurrence, Table 1). Predictions by region are made while other significant predictor variables are standardized (see Section 2). Too few data were available to reliably predict probabilities for Thames or Southern regions; pairwise differences were not tested for significance due to high likelihood of error due to multiple testing. (a) Gray dots indicate locations where otter samples were sourced (note that the distribution of points is not homogenous within regions, particularly NW and Midlands); white lines indicate regional boundaries, which follow river catchments. Taxa are labelled as underlined: *Cyp*rinid, *Sal*monid, *Amp*hibian, and *Cru*stacean. (b) Standard compass point abbreviations are used for NW, NE, and SW Regions, or as underlined for *Wal*es, *Mid*lands, and *Ang*lian). Note adjusted scale on *y*‐axis for Crustacea. Details for mammal predation not shown due to a low probability of occurrence and large standard errors

Region proved to be an important variable for explaining the prevalence of amphibian, crustacean, cyprinid, and salmonid remains and appeared in all top models for these taxa (RI = 1). Amphibians were prevalent primarily in the north and Wales, salmonids primarily in the northwest, and cyprinids, although prevalent in all regions, show much higher prevalence in Anglian Region than elsewhere. Crustacean consumption was rare overall, but was higher in the northwest (Figure 5).

Marine prey were less likely to occur with increased distance from the coast (*z* = 2.56, RI = 1, *p* = .011). Model predictions indicate a very sharp drop in probability of marine prey items to near zero by 50 km. The same relationship was found for eel remains (*z* = 5.29, RI = 1, *p* < .001). Model predictions suggest the probability of recovering eel remains was 0.17 (±0.066) 10 km from the coast, decreasing to 0.073 (± 0.036) by 50 km inland. The reverse was true for amphibian (*z* = 2.843, RI = 1, *p* = .005) and bullhead (*z* = 6.58, RI = 1, *p* = <.001) remains. Amphibian and bullhead remains were predicted at 0.33 (±0.14) and 0.16 (±0.041) 10 km from the coast and 0.44 (±0.16) and 0.23 (±0.055) 50 km inland, respectively.

Over time, the occurrence of eels in otter diet declined significantly (*z* = 2.75, RI = 0.5, *p* = .006). For example, for eels predicted probabilities were 0.35 (±0.14) in 1995 but had dropped to 0.090 (±0.044) by 2010 (Figure 1b). For bullhead and stickleback remains, the converse was true (bullhead: *z* = 2.23, RI = 0.5, *p* = .026, stickleback: *z* = 1.63, RI = 1, *p* = .1), with predicted probabilities of 0.084 (±0.036) in 1995 and 0.22 (±0.055) in 2010 for bullhead (Figure 1c), and 0.073 (±0.026) in 1995 and 0.21 (±0.061) in 2010 for stickleback (Figure 1d).

In models run separately for females, reproductive condition was either not significantly associated with the prey prevalence (amphibian, bullhead, crustacean, cyprinid, eel, insect, salmonid, or stickleback) or it was not possible to test for the importance of reproductive condition due to low sample sizes when males were excluded (for avian, mammal, and marine prey).

## 4 DISCUSSION

Otters are generalist predators that can opportunistically switch their diet according to the availability of prey (e.g., Almeida et al., 2012; Britton et al., 2006; Krawczyk et al., 2016; Remonti, Prigioni, Balestrieri, Sgrosso, & Priore, 2010). Indeed, their generalist tendencies are likely to facilitate their wide global distribution (Clavero, Prenda, & Delibes, 2003; Roos et al., 2015) and have likely assisted their population recovery from widespread declines in 1960–70s. Today, otter populations in the UK are widely assumed to be stable or increasing (Mathews et al., 2018). However, by investigating both biotic and abiotic variables in a long‐term study of otter diet, this study identifies costs associated with prey switching, and reveals potentially differing vulnerabilities between demographic groups and regions of the UK. Such costs and vulnerabilities may impact the persistence of otter populations in the long‐term and reduce their perceived resilience to the degradation of fish diversity and other aquatic prey due to factors such as climate change (Ficke, Myrick, & Hansen, 2007).

### 4.1 The prevalence of empty stomachs

Overall, evidence for recent feeding by otters (i.e., the presence of any prey remains in the gut) declined over time, suggesting that otters were more often going hungry in the latter years of the study. Empty guts were more often seen in individuals found nearer the coast, suggesting either lower prey availability in coastal habitats or behavioral differences—for example, reduced cover or increased anthropogenic disturbance in coastal areas may contribute to reduced feeding. Coastal areas of Wales and England were apparently some of the last to see otter populations return (Crawford, 2010). Although prey type varied both seasonally and regionally, there was no evidence for seasonal or regional variation in the overall occurrence of prey presence versus empty guts, suggesting that otters can be highly effective predators year‐round, across England and Wales. Juvenile otters were approximately seven times more likely to have empty stomachs than both adults and subadults. This is likely to be driven by a combination of factors including having been abandoned/orphaned prior to independence (a likely bias in our sample); being fed largely on milk or muscle tissue which would remain undetected in the morphological analysis of stomach contents; or an increased gut transit time. Model predictions based around the length:condition interaction term suggest that in larger otters, empty stomachs were associated with a long‐term nutritional deficit resulting in loss of condition, whereas in smaller otters this was not the case.

### 4.2 Potential costs and benefits of prey switching

Selection of prey is presumed to reflect a balance between the energetic costs of prey capture and nutritional benefits, following optimal foraging theory (Sih & Christensen, 2001). Eels are often cited as a highly favored prey of otters (e.g., Britton et al., 2006; Jenkins & Harper, 1980; Miranda, Copp, Williams, Beyer, & Gozlan, 2008) due to their high calorific value compared to other prey species (Kruuk, 1995). In recent years, eel population declines have been catastrophic (Westerberg et al., 2018), and reduced eel consumption by otters is reflected in the current study as well as previous, more localized studies (Almeida et al., 2012; Copp & Roche, 2003; Kruuk, 2014). Our study confirms a positive association between eel (and salmonid) consumption and otter body condition, which is not evident for other prey types. Our models can only provide evidence for association, and do not allow us to separate cause and effect. This association may therefore indicate that otters in better condition are able to catch more eels and salmonids, or that consumption of these fat‐rich prey species contributes to improved body condition. Both are likely, and as eel availability continues to decline, body condition is likely to suffer. Our evidence adds weight to previous concerns about food limitation causing localized otter population declines, which may go undetected in broad national distribution surveys (Kruuk, 2014). In our study, the only prey taxa to have increased in otter diet concurrently with eel declines were the bullhead and stickleback. Although these small species were not negatively associated with body condition, there were also no positive associations, suggesting that they are inadequate replacements for eel in nutritional terms. Conversely, this prey switch may reduce otter exposure to persistent, bioaccumulative, toxic chemicals (PBTs) including PCBs (polychlorinated biphenyls) and organochlorine pesticides linked to reproductive potential (Jepson et al., 2016; Sonne et al., 2009) and believed to have caused otter population declines (Chanin, 2003). These chemicals are lipophilic, and consumption of fat‐rich, long‐lived eels is likely to have been a significant exposure route for otters. It is clear, however, that variation in diet must be carefully considered when evaluating apparent differences in pollutant exposure.

Mammals, crustaceans, and amphibians were previously reported as “buffer prey” taken by otters when seasonally abundant, or in the absence of more nutritious prey (Clavero et al., 2003; Jedrzejewska, Sidorovich, Pikulik, & Jedrzejewski, 2001; Krawczyk et al., 2016; Remonti et al., 2010). Both amphibians and crustaceans can form a very significant component of otter diet (Britton et al., 2006; Krawczyk et al., 2016; Parry, Burton, Cox, & Forman, 2011), and it has previously been suggested that ease of capture of amphibians during their congregation for spawning or hibernation may compensate for lower nutritional value in comparison with fish (Krawczyk et al., 2016; Nelson & Kruuk, 1997). The current study, however, suggests a negative association between amphibian consumption and body condition, indicating that reduced energy expenditure due to ease of capture is insufficient to entirely compensate for reduced nutritional value. It should be noted, however, that this statistical association does not reveal whether lower quality prey leads to poorer body condition, or whether otters in poorer condition are less likely to compete for and catch higher quality prey types; both are likely.

### 4.3 Dietary variation across demographic groups

Although only eel and salmonid prey showed a positive association with body condition, this does not mean that other prey species are unimportant. Crustaceans were more likely to be preyed on by smaller otters, probably as a consequence of crustaceans being easier to catch by inexperienced individuals. For subadult otters, crustacean consumption was associated with an improved body condition. This prey type, despite being inferior in terms of nutrition to eel and salmonid, is nonetheless particularly important for population subsets. Age‐related dietary shifts such as these may arise as a consequence of body size or development (e.g., musculature; Bolnick et al., 2003), behavioral changes such as learning (Estes, Riedman, Staedler, Tinker, & Lyon, 2003), or exclusion by larger adults from territories where higher quality prey are abundant. This is in accordance with direct observations from the Scottish island of Mull where only subadults were seen foraging on crustaceans (Watt, 1993). Although juvenile otters were excluded from our analyses, in the four cases where identifiable remains were found in juveniles, two contained mammalian and one, crustacean, remains. For amphibian consumption, associations with length and age‐class suggest a nonlinear association with age: Consumption by subadults becomes more common as they increase in body length (perhaps as a result of learnt behavior patterns) while in adult otters, consumption decreases with body length (perhaps as a result of switching to better quality prey in older individuals).

Dietary differences between the sexes are often attributed to sexual dimorphism, breeding behavior (Begg, Begg, Du Toit, & Mills, 2003), or differing energetic requirements associated with reproduction. Direct observations of otters on Shetland found that males took prey that was 22% larger than prey taken by females without cubs, but that mothers with cubs took prey similar in size to that of males (Kruuk et al., 1987). The current study showed no associations between reproductive condition of females and either presence or type of prey, and showed limited evidence for sex differences (we showed sex differences in cyprinid consumption, but interpretation of this with respect to prey size is not feasible due to the lack of species resolution in this prey family).

### 4.4 Spatiotemporal variation in diet

Individual and demographic variation in prey selection inevitably occurs against a background of spatial and seasonal variation in availability, as well as long‐term changes over time. Complexity escalates when one considers not only absolute availability but also relative availability, across multiple potential prey species, each of which has differing costs and rewards (Ratcliffe, Adlard, Stowasser, & McGill, 2018). Several studies have compared otter diet with indices of prey availability and demonstrate clear associations (e.g., Grant & Harrington, 2015; Remonti et al., 2010). In the current study, a lack of high‐resolution spatiotemporally explicit records of fish (or other species) abundances across England and Wales, over the required fifteen‐year period, precludes such explicit comparisons. Broad patterns in both spatial and seasonal variation, however, do support presumed associations with availability. For example, the distinct trough in amphibian consumption in summer is at a time when amphibians are away from their aquatic breeding areas, and the largest peak coincides with the amphibian spring breeding migration (Beebee, 2013). Similar seasonal trends in amphibian consumption have been shown previously in dietary studies of limited geographic range (e.g., Britton et al., 2006; Clavero et al., 2003; Parry et al., 2011). Secondary peaks (although predicted with less confidence) are indicated in autumn and winter, potentially reflecting an autumn submigration, and winter predation at a time when amphibians are vulnerable due to inactivity (Beebee, 2013). Eel consumption was most common near the coast and peaked in May followed by a gradual decline—presumably reflecting the mass arrival of elvers in UK estuaries in around April each year (White & Knights, 1997). Bullhead were most frequently taken inland (perhaps reflecting their tendency to inhabit small, upland streams (Maitland & Campbell, 1992)) and in autumn. An autumn peak in bullhead consumption is contrary to an earlier UK study where bullhead consumption peaked in summer (Grant & Harrington, 2015). Grant and Harrington (2015) suggest that otters were switching between consuming bullhead in summer and cyprinids in winter, but their study was restricted to one river in southern England, whereas the current study indicates the reverse. Interpreting temporal trends for cyprinids should be treated with caution given the ambiguity in species identification, but a winter peak (December‐February) is in accordance with other European studies based on the analysis of spraints (Breathnach & Fairley, 1993; Chanin, 1981; Grant & Harrington, 2015; Taastrom & Jacobsen, 1999) and is likely to reflect increased ease of capture with reduced fish swimming speeds in colder water temperatures. The summer peak evidenced in the current study may reflect ease of capture due to shoaling behavior, which occurs variously from April to June (Maitland & Campbell, 1992). Where regional variation was significant, cyprinid prey were particularly important in Anglian Region, whereas salmonids were important in the northwest, and amphibians in the north and Wales.

## 5 SUMMARY

The current study supports the theory that otters in Wales and England are generalist predators with the capacity to prey switch. Abiotic associations are largely consistent with previous studies and expectations, while the biotic data collected at postmortem have allowed us to explore some of the potential costs and benefits of prey switching. These associations are important aspects of carnivore foraging strategies and population dynamics, and are a key consideration for conservation management in a changing world.

## CONFLICT OF INTEREST

None declared.

## AUTHOR CONTRIBUTION

**Rosemary J. Moorhouse‐Gann:** Formal analysis (lead); Writing‐original draft (lead). **Eleanor F. Kean:** Data curation (supporting); Formal analysis (supporting); Supervision (supporting); Writing‐review & editing (supporting). **Gareth Parry:** Investigation (supporting); Methodology (supporting); Writing‐review & editing (supporting). **Sonia Valladares:** Investigation (supporting); Writing‐review & editing (supporting). **Elizabeth A. Chadwick:** Conceptualization (lead); Data curation (lead); Formal analysis (supporting); Funding acquisition (lead); Project administration (lead); Supervision (lead); Visualization (lead); Writing‐review & editing (lead).

## Data Availability

Dietary, biotic, and abiotic data are archived on Dryad: https://doi.org/10.5061/dryad.5dv41ns3k.

## Appendix 1

**TABLE A1:** Results of generalised additive models from Model Groups 1 and 2, both modelling the prevalence of empty stomachs

Model group	Variable	Estimate	*SE*	*Z* value	Pr(>|*z*|)	Relative variable importance
1	Age Class = Juvenile	5.63E+00	1.87E+00	3.01	0.00261**	—
1	Age Class = Sub‐adult	4.01E−01	6.12E−01	0.654	0.51296	—
1	Cause of death = Sudden	−2.53E+00	8.45E−01	−2.987	0.00282**	—
1	Cause of death = Unknown	2.86E+00	3.47E+00	0.825	0.40918	—
1	Body condition	2.18E+01^a^	9.84E+00	2.217	0.02663*	—
1	Body length	2.65E−02^a^	9.59E−03	2.767	0.00566**	—
1	Distance from the coast	−1.75E−05	7.22E−06	−2.426	0.01528*	—
1	Sex = Male	1.92E+00	7.77E−01	2.477	0.01326*	—
1	Year	4.53E−01	1.75E−01	2.59	0.0096**	—
1	Body condition:Body length	−2.40E−02	9.64E−03	−2.494	0.01265*	—
2	Reproduction status	—	—	—	—	0.4

Note

For Model Group 1, there was only one top model so a model averaging approach was not applied and the results displayed are for the single top model. Model Group 2 (females only) revealed that reproductive status was not an important predictor of empty stomachs (shown) so the remainder of the results for Model Group 2 are not shown.

^a^ To be interpreted in interaction term.

**TABLE A2:** Results of averaged generalised additive models from Model Groups 3 and 4, each model modelling the prevalence of a single prey type

Model group	Variable	Prey type										
		Amphibian	Avian	Bullhead	Crustacean	Cyprinid	Eel	Insecta	Mammal	Marine	Salmonid	Stickleback
3	Age class	1	0.46	0	1	0.37	0.44	0.58	1	0.1	0.75	0.09
3	Sex	0.57	0.19	1	0	1	0.13	0.42	1	0.11	0	0.38
3	Body condition	0.85 (*)	0.71	0	1	0.65	1 (*)	0.68	1 (*)	0.29	1 (*)	0
3	Body length	1 (*)	0.14	1 (*)	1	0.39	0.5	0.46	0.43 (.)	0.11	1 (*)	0.89 (.)
3	Sex: Age class	0.26	0.06	0	0	0	0.13	0.35	1	0	0	0
3	Sex: Body condition	0	0.06	0	0	0.05	0	0.15	0	0	0	0
3	Sex: Body length	0.45	0	0.45	0	0	0	0	0	0	0	0
3	Age class: Body length	1	0	0	0	0	0.29	0.35	0.34	0	0.53	0
3	Age class: Body condition	0.18	0.06	0	1	0.09	0	0	0.34	0	0.21	0
3	Body condition: Body length	0	0	0	0	0.08	0	0	0	0	0	0
3	Month	1	0	1	1	1	1	0.58	0	0	0.8	0
3	Year	0.5 (.)	0.11	0.5 (*)	0.5	0.5	0.5 (**)	0.32	0	0.25	0.6 (.)	1
3	Region	1	0	0	1	1	0	0	1	0	1	0.15
3	Distance from coast	1 (+,**)	1 (−)	1 (***)	1	0.89	1 (***)	0.33	0	1 (*)	0	0.23
4	Reproductive status	0	NA	0	0	0	0	0	0	NA	0	0

Note

Relative variable importance (RI) is shown for each variable, for each modelled prey type, significance (*p*) is indicated as: *<.05, **<.01, ***<.001. Model Group 4 (females only) revealed that reproductive status was not an important predictor of the prevalence of prey types (although note that for some prey types [marked NA] models were not run due to small sample sizes when males were removed). The remainder of the variables for Model Group 4 are therefore not shown (e.g., age class, sex, etc) as these were all incorporated in model group 3.
